# Draft Genome Sequence of the Cellulolytic Fungus Chaetomium globosum

**DOI:** 10.1128/genomeA.00021-15

**Published:** 2015-02-26

**Authors:** Christina A. Cuomo, Wendy A. Untereiner, Li-Jun Ma, Manfred Grabherr, Bruce W. Birren

**Affiliations:** a Broad Institute of Harvard and MIT, Cambridge, Massachusetts, USA; b Brandon University, Brandon, Manitoba, Canada; c University of Massachusetts, Amherst, Massachusetts, USA; d Science for Life Laboratory, Department of Medical Biochemistry and Microbiology, Uppsala University, Uppsala, Sweden

## Abstract

Chaetomium globosum is a filamentous fungus typically isolated from cellulosic substrates. This species also causes superficial infections of humans and, more rarely, can cause cerebral infections. Here, we report the genome sequence of C. globosum isolate CBS 148.51*,* which will facilitate the study and comparative analysis of this fungus.

## GENOME ANNOUNCEMENT

Chaetomium (family *Chaetomiaceae*, class *Sordariomycetes*, phylum *Ascomycota*) is a genus of filamentous fungi encompassing species important in the decomposition of plant and other cellulose-rich materials that can be isolated easily from dung, plant debris, and soil. Chaetomium includes thermophilic species capable of growth at elevated temperatures as well as a few species that cause infections in vertebrates. Chaetomium globosum, the type species of the genus, is commonly isolated from decaying plant material, seeds, and other cellulosic substrates. Of the more than 150 species of Chaetomium described to date, C. globosum is the most frequently isolated and inhabits the widest range of environments (1).

Chaetomium globosum is a medically important fungus and is encountered typically as an agent of skin and nail infections in humans. This species more rarely causes cerebral and systemic infections, but such mycoses have high mortality rates, particularly in immunocompromised patients (2). Chaetomium globosum is important to human health as a contaminant in indoor environments since it is known to produce mycotoxins (3, 4) and act as an allergen.

The sequenced strain CBS 148.51 was isolated from stored cotton in Washington, DC. This strain is commonly used in testing paper and polymers for fungal resistance (5). Three size-selected libraries were constructed from genomic DNA. These included a 4-kb plasmid, a 10-kb plasmid, and a 40-kb fosmid library; each library was paired-end sequenced using Sanger technology. The resulting 568,566 reads were assembled using Arachne version 3.0, with an average of 8.9× sequence depth in the final assembly.

Based on the assembly, the genome size was estimated to be 34.3 Mb with a GC content of 55.6%. The assembly was organized in 1,245 contigs, which are linked by paired-end reads into 37 scaffolds. The average base is found in a scaffold of *N*_50_ size 4.72 Mb and a contig of *N*_50_ size 50.76 kb. The assembly is highly contiguous; the largest 8 scaffolds account for 98% of the assembly bases. For gene prediction, Fgenesh, Fgenesh+, and Genewise, which were previously trained for the related species Neurospora crassa (6), were used. A total of 11,124 protein-coding genes were predicted by aligning publicly available expressed sequence tags to the genome and combining those alignments with the output from Fgenesh, Fgenesh+, Genewise, and GeneId. A total of 5.57% of the assembly was identified as interspersed repeats using RepeatMasker (http://www.repeatmasker.org) to identify copies of *de novo* repeats identified by RepeatModeler (http://www.repeatmasker.org/RepeatModeler.html) and fungal sequences from RepBase (7).

This genome sequence of C. globosum will serve as an important reference for further studies of the basis of its cellulose specificity, for genes that enable human infection and for further comparative studies with other fungi.

### Nucleotide sequence accession number.

The whole-genome sequence and annotation of C. globosum isolate CBS 148.51 (ATCC 6205) have been deposited at DDBJ/EMBL/GenBank under the accession number AAFU00000000.
